# Quantitative Monitoring of the *Chlamydia trachomatis* Developmental Cycle Using GFP-Expressing Bacteria, Microscopy and Flow Cytometry

**DOI:** 10.1371/journal.pone.0099197

**Published:** 2014-06-09

**Authors:** François Vromman, Marc Laverrière, Stéphanie Perrinet, Alexandre Dufour, Agathe Subtil

**Affiliations:** 1 Institut Pasteur, Unité de Biologie des Interactions Cellulaires, Paris, France; 2 Centre National de la Recherche Scientifique, URA 2582, Paris, France; 3 Université Pierre et Marie Curie, Cellule Pasteur UPMC, Paris, France; 4 Institut Pasteur, Unité d’Analyse d’images biologiques, Paris, France; University Freiburg, Germany

## Abstract

Chlamydiae are obligate intracellular bacteria. These pathogens develop inside host cells through a biphasic cycle alternating between two morphologically distinct forms, the infectious elementary body and the replicative reticulate body. Recently, *C. trachomatis* strains stably expressing fluorescent proteins were obtained. The fluorochromes are expressed during the intracellular growth of the microbe, allowing bacterial visualization by fluorescence microscopy. Whether they are also present in the infectious form, the elementary body, to a detectable level has not been studied. Here, we show that a *C. trachomatis* strain transformed with a plasmid expressing the green fluorescent protein (GFP) accumulates sufficient quantities of the probe in elementary bodies for detection by microscopy and flow cytometry. Adhesion of single bacteria was detected. The precise kinetics of bacterial entry were determined by microscopy using automated procedures. We show that during the intracellular replication phase, GFP is a convenient read-out for bacterial growth with several advantages over current methods. In particular, infection rates within a non-homogenous cell population are easily quantified. Finally, in spite of their small size, individual elementary bodies are detected by flow cytometers, allowing for direct enumeration of a bacterial preparation. In conclusion, GFP-expressing chlamydiae are suitable to monitor, in a quantitative manner, progression throughout the developmental cycle. This will facilitate the identification of the developmental steps targeted by anti-chlamydial drugs or host factors.

## Introduction

Chlamydiae are obligate intracellular bacteria that grow in very diverse eukaryotic hosts, including humans. *Chlamydia trachomatis* is the most prevalent sexually transmitted bacterial pathogen (more than 2.5 million infections annually in the United States as estimated by the Center for Disease Control in 2012) and can lead to severe pathologies including infertility, ectopic pregnancy, and pelvic inflammatory disease. Conjunctival inflammation as a result of *C. trachomatis* infection is the leading cause of blindness by an infectious agent, with about 8 million people irreversibly visually impaired by trachoma and an estimated 84 million cases in need of treatment (World Health Organization 2011) [1].

Chlamydiae develop in a biphasic cycle, which is a landmark of the order [2]. The infectious forms of the bacteria, called elementary bodies (EBs), are characterized by a small size (around 0.3 µm), a rigid cell wall, densely packed DNA, and reduced metabolic activity. Upon entry into a host cell, typically an epithelial cell, EBs convert to reticulate bodies (RBs). RBs are larger (1–2 µm), metabolically active, and multiply within a membrane-bound vacuole called the inclusion. After several rounds of division, RBs convert back to the EB form, still within the inclusion, before ultimately exiting the host cell. Completion of the whole cycle takes two or more days depending on the species. The initial steps of infection (adhesion, entry, conversion, and division) are asynchronous, ultimately leading to a population of infected cells with inclusions of variable sizes. These inclusions typically contain a mixture of EBs and RBs at later times during infection.

The obligate intracellular growth of chlamydiae, and the absence of genetic manipulation tools, have limited the development of tools to measure the progression of the infectious cycle through its different steps *in vitro*. Adhesion can be quantified by flow cytometry using antibodies against the major component of the outer membrane (MOMP) [3] or using bacteria labeled with a fluorescent dye [4]. To measure bacterial entry, the method of choice is microscopy, using a two-step permeabilization protocol to distinguish intracellular from extracellular bacteria [5], [6]. This method is time consuming and requires intensive work to be precise. Intracellular growth is usually assessed by quantitative PCR, measuring genome number [7], or by microscopy, measuring inclusion number and size. Alterations in the infectious cycle affect the number of infectious particles produced, and the “infectious progeny” is enumerated through re-infection assays and quantification of the inclusion forming units (IFUs) by microcopy. This requires fixation of the samples followed by manual or automatic counting of the inclusions by microscopy after inclusion staining with anti-bacterial antibodies [8], [9]. Various methods for staining and directly counting EBs under the microscope have also been described, yet all are rather tedious and rarely employed [10].

Recently, following the pioneering work by the Clarke lab, *C. trachomatis* strains stably expressing fluorescent proteins were obtained in various laboratories [11]–[13]. Bacteria expressing the green fluorescent protein (GFP) are of particular interest because all fluorescent microscopes and flow cytometers are equipped with the lasers and filters required to detect this fluorochrome. Here, we show that the fluorescent signal of GFP-expressing chlamydiae allows monitoring of the progression through the developmental cycle in a quantitative manner. This has several advantages over current methods. Thousands of events can be rapidly analyzed by flow cytometry, generating highly quantitative data even on rare events. Calibration of the instrument allows detection of individual fluorescent EBs and thus direct counting of the particles. The fluorescence level in individual EBs is also sufficient for detection by microscopy, and we show that automated tools allow for rapid quantification of bacterial entry into cells. Using these quantitative tools, the action of anti-chlamydial compounds on different steps of the chlamydial developmental cycle can easily be assessed.

## Materials and Methods

### C. trachomatis strains

One clonal population from *C. trachomatis* L2 strain 434 (ATCC) was plaque isolated before transformation with SW2::GFP [11] or p2TK2-SW2 IncDProm-RSGFP-IncDTerm [13] as has been previously described [11]. EBs were purified on a density gradient as described [14].

### Cell culture, transfection and chemicals

HeLa cells were cultured in Dulbecco’s modified Eagle’s medium with Glutamax (DMEM, Invitrogen) supplemented with 10% (v/v) fetal calf serum (FCS). Construction of the histidine-tagged IncA constructs was described elsewhere [15]. Cells were transfected 24 hrs after seeding using JetPrime transfection kit (Polyplus transfection) and infected 24 hrs later. Tetracycline (12.5 mg/ml stock in ethanol) and cytochalasin D (5 mg/ml stock in DMSO) were purchased from Sigma and stored at −20 °C.

### Quantitative-PCR analysis

Cells were infected at a multiplicity of infection (MOI) between 0.1 and 0.2 for each of the three strains tested. At the indicated time points, cells were gently detached using 0.5 mM EDTA in PBS, pelleted by centrifugation at 400x*g*, resuspended in PBS, and stored at −20 °C. DNA was extracted using the DNeasy blood & tissue kit (Qiagen) following the manufacturer instructions, with cell lysis at 65 °C for better bacterial lysis. Total DNA concentrations were measured and normalized to 50 ng/µl using a Nanodrop spectrophotometer (Thermo Scientific). Note that the contribution of bacterial DNA to total DNA is negligible compared to host DNA in these experimental conditions. Primers targeting the *ompA* gene (GGTTTCGGCGGAGATCCT and AGTAACCAACACGCATGCTGAT) were used at 10 µM with the Quantitect SYBRgreen PCR kit (Qiagen) following the manufacturer’s instructions. Q-PCR reactions were run in an LC480 Lightcycler thermocycler (Roche), starting with an activation phase of 15 min at 95 °C, followed with 40 cycles with an annealing temperature of 54 °C. Data were normalized to standard curves of purified L2 genomic DNA amplified in parallel with the experimental samples.

### Observation of fluorescent EBs

L2 or L2^incD^GFP EBs were centrifuged at 400x*g* on poly-L-lysine treated coverslips and fixed for 30 min in 4% paraformaldehyde (PFA) 120 mM sucrose in phosphate buffered saline solution, PBS (fixation buffer). Bacteria were stained with a mouse anti-MOMP-LPS (Argene #11–114) antibody followed with Cy5-conjugated secondary antibodies. Bacteria were permeabilized for 15 min in 0.3% (v/v) Triton X-100 in PBS 1 mg/ml BSA prior to DNA staining for 30 min using 0.5 µg/ml Hoechst 33342 (Molecular Probes) in PBS with 1 mg/ml BSA. Coverslips were then mounted in a Mowiol solution.

### Quantification of entry with a semi-automated procedure

Entry experiments were performed on cells seeded the day before on coverslips (40,000 cells/well) in 24-well plates. Prior to infection, cells were incubated at 37 °C for 30 min in culture medium supplemented or not with 1 µg/ml cytochalasin D (Sigma) or DMSO and were maintained in this buffer until fixation. To disrupt bacterial aggregates, EBs purified on a density gradient [14] were briefly sonicated prior to infection. Cells were incubated at 4 °C for 15 min in DMEM 10% FCS before adding the bacteria (MOI = 10) for another 30 min at 4 °C. Medium was replaced by medium prewarmed at 37 °C, and plates were transferred to the 37 °C incubator for the indicated times before fixation in ice-cold fixation buffer for 30 min. Extracellular bacteria were stained with a mouse anti-MOMP-LPS as described above. Pictures of fields with 5–10 cells were acquired using an Axio observer Z1 microscope equipped with an ApoTome module (Zeiss, Germany) and a 63× Apochromat lens. Pictures were taken with a Coolsnap HQ camera (Photometrics, Tucson, AZ) using the software Axiovision. A minimum of 80 bacteria was analyzed per condition. We designed an automatic, ready-to-use analysis protocol for the Icy software [16] to perform the quantification on entire image folders without manual intervention (The Chlamentry protocol will be made publicly available on the Icy website upon publication). First, a wavelet-based detection module [17] was used to detect all objects in the green and red channels. Then, an object-based colocalization module was used to visualize and quantify the colocalization between the two detection sets. Two detections were considered colocalized under a distance threshold of 4 pixels (i.e. 400 nm) between their center of mass, accounting for the chromatic aberration of the imaging setup. Finally, the protocol produced a comprehensive result sheet containing the number and location of detected objects in each channel, the number of colocalized detections (i.e. number of extracellular bacteria), and a final script calculated the ratio of [green - colocalized detection] to [green detection] (i.e. ratio of internalized bacteria). Of note, the light sonication procedure preceeding infection can lead to the appearance of red-and-not-green dots. These red dots are also usually not visible in the blue channel (DNA), and presumably correspond to bacterial wall debris. We used conditions where such events represented less than 10% of the total red staining. In addition, these objects are not scored by the software since they are not green, and therefore do not affect the measured efficiency of entry.

### Flow cytometry on infected cells

Cells were seeded in 6-well plates (400,000 cells/well) the day before infection. Cells were infected for one hour with L2^incD^GFP EBs purified on a density gradient at the indicated MOI before changing the medium and returning the plate to the incubator. At the indicated times, cells were washed with PBS and gently detached using 0.5 mM EDTA in PBS. Samples were fixed in PFA 2% in PBS and stored over-night at 4 °C. For adhesion experiments, cells were incubated for 4 hrs at 4 °C with L2^incD^GFP EBs at the indicated MOI before being washed, detached and fixed as described above. Flow cytometry analysis was performed with a FACS Gallios (Beckton Coulter) using the FL-1 (detecting fluorescence emission between 505 and 545 nm), the FSC (relative cell size) and the side scatter detectors (cell granulometry or internal complexity) on 1/10 of the sample diluted in PBS. The FACS Gallios parameter FSC collection angle N (Narrow FSC angles 1 – 8°) was used, triggering on the FSC channel during acquisition. A minimum total of 10,000 gated events were collected for each sample. Data were analyzed using the Kaluza 1.2 software (Beckman Coulter) and FlowJo. Calibration beads were purchased from Spherotech.

For analysis of transfected cells, after fixation the cells were centrifuged at 1500x*g*, washed in PBS, centrifuged again, and incubated for 1 hr with home-made rabbit anti-histidine tag antibodies in 1 mg/ml BSA, 0.05% (w/v) saponin in PBS. The cells were washed and incubated for one hour in the same buffer with anti-rabbit antibodies conjugated to Cy5. The cells were washed, resuspended in PBS and analyzed by flow cytometry in the FL-1 (green) and FL-4 (far-red) channels.

### Flow cytometry on EBs

EBs purified on a density gradient were serially diluted and fixed in 2% PFA in PBS. Flow cytometry analysis was performed with a FACS Gallios using the FL-1, the FSC, and the side scatter detectors on 1/10 of the sample diluted in PBS. The W2 (Enhanced Wide angle) was used for the FSC parameter collection, triggering on the FL-1 channel during the acquisition, 100,000 events were acquired. Serial dilutions of EBs freshly prepared (no freezing step) were used, on one hand to quantify particle concentration by flow cytometry, and, on the other hand to infect fresh HeLa cells and determine the titer expressed as IFU/ml by serial dilutions, as has been described [14].

## Results

### GFP is detected both in RBs and EBs

**Figure 1:**
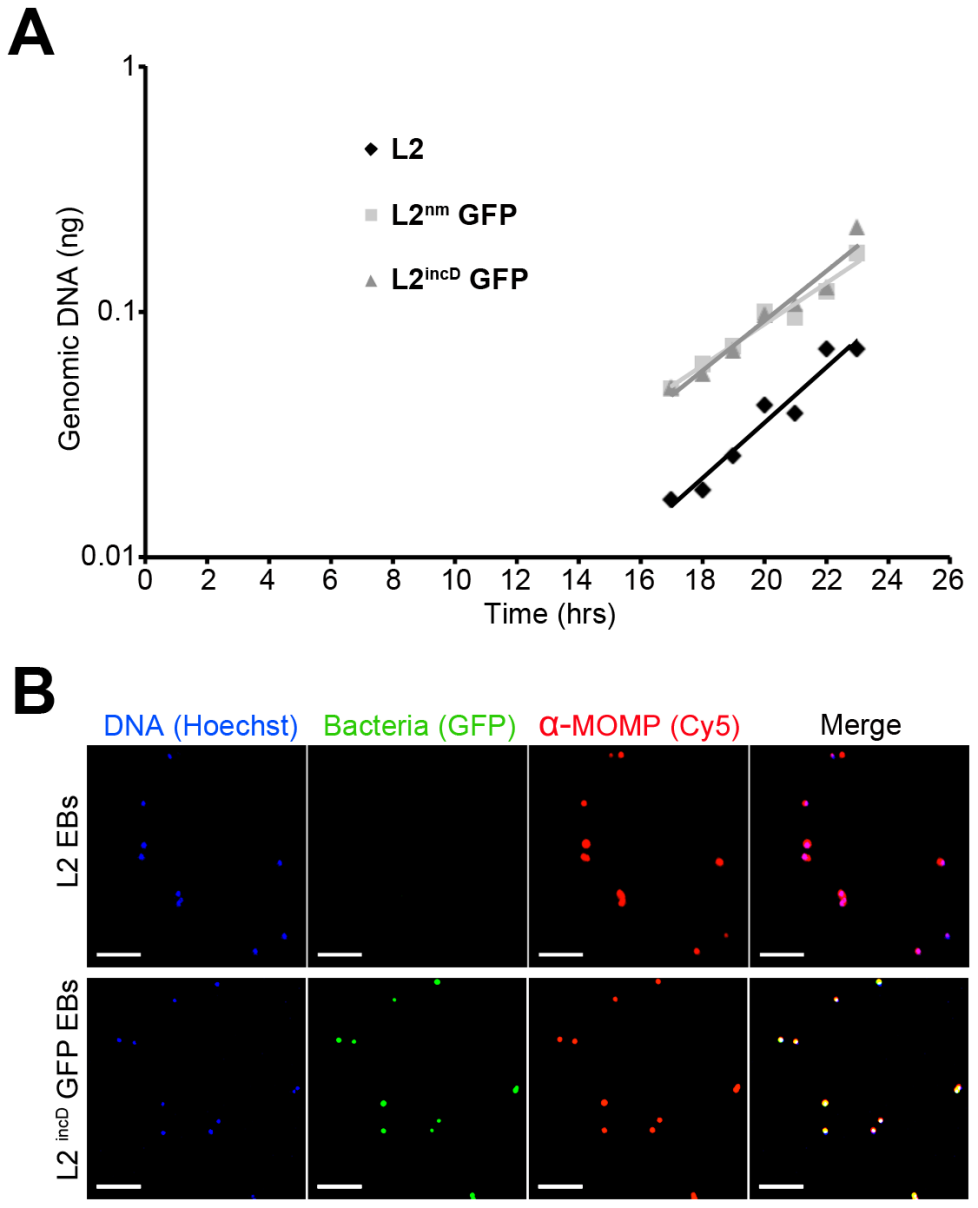
GFP-expressing *C. trachomatis* serovar L2 strains are suitable to track RBs and EBs. (A) Genomic DNA accumulation over time for the parental strain and the two GFP-expressing strains, L2^nm^GFP and L2^incD^GFP, with GFP expression controlled by a neisserial (nm) promoter or the promoter for the chlamydial *incD* gene, respectively. Bacterial DNA was quantified by Q-PCR on the *ompA* gene. An experiment representative of three is shown. In this experiment, the MOI for the parental strain was about 2 to 3 times less than that for the two other strains, accounting for the lower bacterial DNA amount at all time points. Nevertheless, the growth curves are similar for the three strains (B) Coverslips were coated with L2 (top) or L2^incD^GFP (bottom) EBs. After fixation, the bacterial envelope was stained with an anti-MOMP/LPS antibody followed with Cy5 coupled secondary antibodies, and bacterial DNA was labeled with Hoechst. The scale bar represents 5 µm.

GFP-expressing *C. trachomatis* will be suitable to monitor infection if they show similar growth rate as the parental strain and if the fluorescent protein is expressed at a level sufficient for detection. We compared the growth kinetics of the parental *C. trachomatis* L2 strain with two different strains obtained by transformation with a plasmid expressing GFP in one case under the control of a promoter derived from *Neisseria meningitidis* [11] and in the second case from the promoter of the *incD* gene of *C. trachomatis* [13]. In the rest of the manuscript, these three strains are designated L2, L2^nm^GFP and L2^incD^GFP, respectively. Bacterial growth was measured by Q-PCR of chlamydial genome content over time between 17 and 23 hrs of infection, which are within the exponential growth phase [7]. Similar growth rates were observed (Fig. 1A), demonstrating that expression of the exogenous gene has no impact on bacterial multiplication. We measured a doubling time of about 3 hrs, a value similar to the doubling time reported for L2 [7].

Fluorescent RBs have been shown to be amenable to microscopy, including live [11], [13], but whether GFP is also present in EBs to a detectable level has not been studied. Purified EBs from the L2^incD^GFP strain were attached to a coverslip using poly-L-lysine, and the bacteria were fixed using PFA and stained with anti-MOMP antibodies followed with secondary antibodies coupled to a red fluorescent dye. Green particles were observed on the coverslip incubated with purified L2^incD^GFP EBs and not with purified L2 EBs (Fig. 1B). These particles correspond to bacteria since they are co-stained with anti-MOMP antibodies and with DNA labelling. Thus, the level of GFP present in EBs is sufficient for detection by microscopy.

Fluorescence of L2^incD^GFP EBs was stronger than that of L2^nm^GFP EBs, a difference that was also observed when the fluorescence of whole inclusions was compared (not shown). This higher expression does not impact growth as infection with L2^incD^GFP resulted in similar infectious progeny as with the parental strain [13]. We therefore chose to use the L2^incD^GFP strain to ask if these bacteria were suitable to monitor the different steps of the developmental cycle using flow cytometry.

### Quantitative measure of the adhesion step by flow cytometry

**Figure 2:**
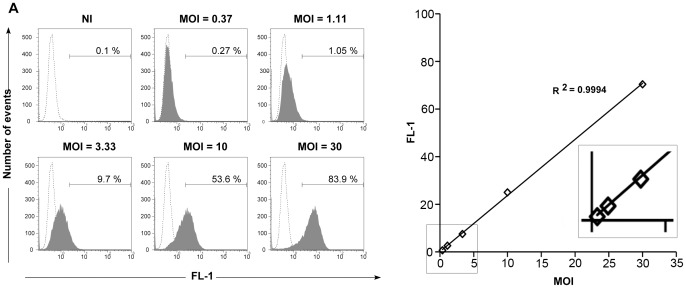
Quantification of *C. trachomatis* adhesion by flow cytometry. Cells were incubated for 4°C with the indicated MOI of L2^incD^GFP purified EBs, before fixation and analysis in the green channel by flow cytometry. (A) Histograms of fluorescence in the green (FL-1) channel. In each panel, the fluorescence of non-infected cells is reported for comparison (dashed line). The horizontal bar delimits the fluorescence above background level and the percentage of cells (out of total cells) reaching these fluorescence values is indicated above. NI  =  non-infected. (B) The mean fluorescence of non-infected cells was subtracted from the mean fluorescence of infected cells and the resulting fluorescence value was plotted against the MOI. The inset shows an enlargement of the values obtained at low MOI. This experiment is representative of two.

We examined whether bacterial adhesion could be measured by flow cytometry. We incubated cells with different amounts of purified L2^incD^GFP EBs for 4 hrs at 4 °C. Cells were then fixed and analyzed by flow cytometry. Even at a low MOI of 1.11, a small shift of the cell-associated green fluorescence was observed (Fig. 2A). The difference in mean fluorescence increased in a linear manner with bacterial concentration up to the highest MOI tested (MOI = 30) (Fig. 2B). Thus, the GFP signal can be used as a convenient read-out to quantify EB adhesion by flow cytometry. The possibility of analyzing a large number of events make this approach quantitative even at low MOI.

### Fluorescence of infectious particles is sufficient for tracking entry by automated microscopy tools

**Figure 3:**
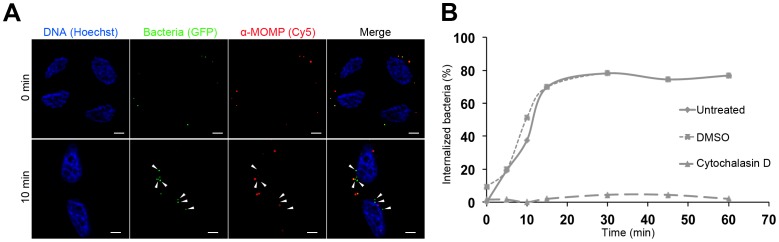
Quantification of *C. trachomatis* internalization by automated microscopy. Cells were pre-incubated at 37 °C for 30 min in culture medium alone (untreated) or supplemented with 1 µg/ml cytochalasin D or solvent (DMSO). Cells were then transfered to 4 °C and incubated with bacteria (MOI = 10) for 30 min. At time zero, the plates were transferred to 37 °C and incubated for the indicated times. The cells were fixed, extracellular bacteria were labeled with anti-MOMP antibodies followed with Cy5-coupled secondary antibodies, and DNA was labeled with Hoechst. (A) Representative fields of the untreated control in the blue (first column), green (second column) and far-red (third column) channels with the merged pictures shown on the right. Prior to transfer to 37 °C (top) all bacteria (green) are extracellular (red), while after 10 min at 37 °C half of the bacteria are internalized (arrowheads). The scale bar represents 5 µm. (B) Kinetics of bacterial entry. Images were processed with the ICY software as described in the methods section. Each time point represents averages on more than 80 bacteria from 10 different fields. One experiment representative of three is shown.

We next asked whether the GFP signal could be used to monitor bacterial entry into cells. For future applications, we anticipated that co-localization with host proteins, or other information on the spatial organization of the entry step, would be sought. We therefore used a microscopy-based approach, which, in contrast to flow cytometry, gives access to spatial information. Cells were incubated with bacteria at 4 °C to allow bacterial attachment but not entry, and then transferred to a 37 °C incubator for variable times. The cells were then fixed with paraformaldehyde, and extracellular bacteria were stained with anti-MOMP antibodies followed with Cy5-conjugated secondary antibodies. For each time points, 10 pictures in randomly selected fields were acquired in the green and the far-red channels and analyzed using the ICY software, as detailed in the Methods. Preliminary experiments done with cells infected for 15 min showed that the same proportion of bacteria was scored intracellular when using this automated procedure or when counting manually. Furthermore, when the bacteria were stained in two steps as previously described [6], similar internalization levels were observed as with the one step procedure described here, validating the new method. We next used this procedure to follow *C. trachomatis* L2 entry over the first hour of infection. As a control we included cells treated with cytochalasin D, a known inhibitor of chlamydial entry [18]–[20], or the solvent DMSO. Around 50 percent of the bacteria were internalized within the first 10 min of incubation at 37 °C and reached a plateau with 75% intracellular bacteria within 30 min (Fig. 3). To the best of our knowledge these kinetics represent the first precise description of the rate of entry of *C. trachomatis*. Our data fit with the reported efficiency of internalization of L2 in HeLa cells within 30 min [5]. As expected, depolymerization of the actin cytoskeleton using cytochalasin D completely abrogated entry. Interestingly, it also reduced bacterial adhesion at 4 °C by about one third (data not shown).

### Quantitative measurement of antichlamydial activities

**Figure 4:**
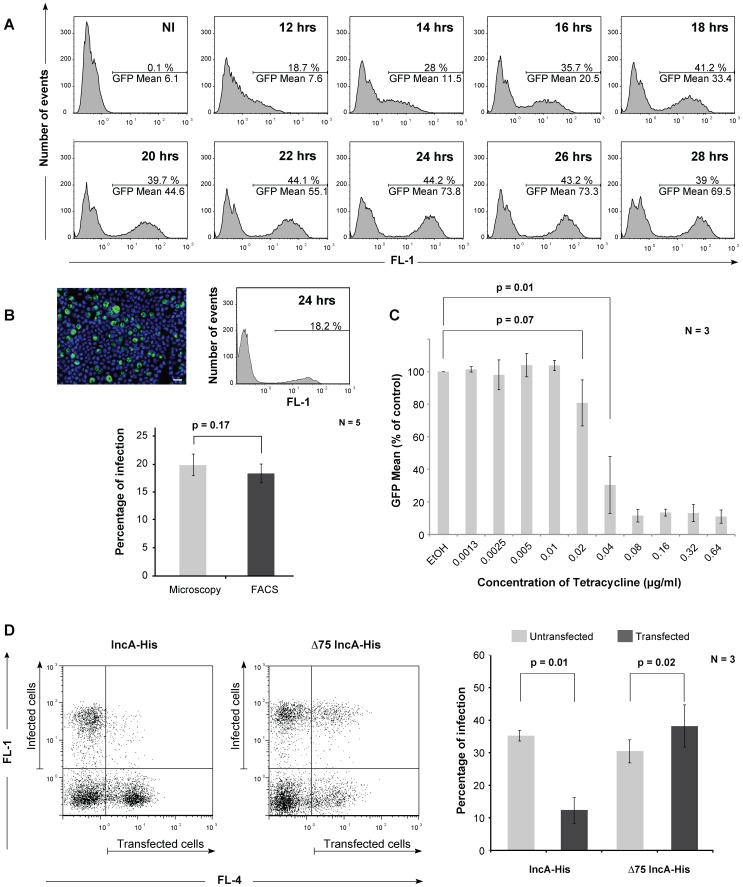
Quantitative measurement of the *C. trachomatis* growth cycle using flow cytometry. (A) Kinetics of chlamydial growth. Cells were infected at a MOI = 0.5 and fixed at the indicated times. Samples were analyzed by flow cytometry as described in the Methods section, and histograms of fluorescence in the green channel (FL-1) are shown. For each time point, 10,000 cells were analyzed. The horizontal bar delimits the fluorescence above background level( = infected cells). The percentage of cells included in this gate, and their mean fluorescence, are indicated. NI  =  non-infected. (B) Comparison of the determination of infection rates by flow cytometry and microsocopy. Cells were infected at MOI = 0.2 in a 36 mm dish with one coverslip in the dish. Twenty-four hours later, cells on the coverslips were fixed and processed for microscopy toma nually count the percentage of infected cells (top left panel, cellular DNA appears in blue and inclusions are green, bar = 20 µm). The rest of the dish was used for determining the infection rate by flow cytometry (top right panel). The average of five measurements with each method is shown. A t-test showed no statistical difference between the two methods. (C) Determination of tetracycline IC-50 by flow cytometry. Cells were infected (MOI = 0.3) for one hour prior to tetracycline addition and incubated for 24 hrs before analysis by flow cytometry as described above. The histogram shows the mean fluorescence in the infected population relative to the value measured in infected cells treated with ethanol only, the error bars show the standard deviation in the triplicate of this experiment. The experiment has been reproduced three times. (D) Analysis of infection rates in a non-homogenous population. Cells were transfected for 24 hrs with the indicated construct prior to infection with L2^incD^GFP. At 24 hrs post-infection, cells were detached and fixed, and the His tag was stained in red as described in the Methods section. Transfected cells were positive in the red channel (FL-4), infected cells in the green channel (FL-1). A dot-plot analysis of the two parameters is shown for cells transfected with IncA-His (left) or Δ75IncA-His (right). For each condition, the percentage of infected cells in the transfected and non-transfected populations is reported in the histogram. The experiment shown is representative of three independent assays with error bars showing the standard deviation. In panels B, C and D results of t-tests are reported.

Bacterial entry is followed by differentiation into the RB form, and intracellular multiplication. We followed the intracellular growth of L2^incD^GFP using flow cytometry. Green bacteria were observed at all time points by microscopy and detected by flow cytometry. At early time points, the GFP signal is too low to fully separate the infected cells from the non-infected ones (Figure S1). This limitation is due to the fact that each infected cell only contains one to a few bacteria, whose GFP signal is within the range of the autofluorescence level of the host cell. The population of infected cells was fully discernible from non-infected cells in the green fluorescence channel 18 hrs post infection (Fig. 4A). As expected, within one infection round, the percentage of infected cells remained stable between 18 hrs and 30 hrs of infection while the mean fluorescence increased as the bacteria divided. We verified that the percentage of infected cells measured by flow cytometry matched the percentage of infection measured by visual examination of the population by microscopy, which is currently the method of choice for quantifying IFUs (Fig. 4B).

The mean intensity of the whole population (including infected and non infected cells) can be used as a practical read-out of the activity of anti-bacterial drugs. To illustrate this application we measured the mean fluorescence in infected cells exposed to increasing concentrations of tetracycline, a potent inhibitor of bacterial growth. We determined the IC_50_ for this antibiotic to be 40 ng/ml (Fig. 4C).

One strong advantage of the single cell analysis lies in the possibility to measure differences in infection rates within non-homogenous populations. For instance, it can show in one simple step whether the overexpression of a particular protein affects the bacterial developmental cycle. As a proof of principle we evaluated the effect of ectopic expression of the inclusion protein IncA on bacterial growth. Cells were transfected with a plasmid expressing His-tagged IncA, either full-length or with a 75 amino acid truncation at its N-terminus. Twenty-four hours later, cells were infected with L2^incD^GFP strain, and 24 hrs later they were fixed and processed for analysis by flow cytometry, with detection of the transfected population in the red channel and of the infected population in the green channel (Fig. 4D). We observed a three-fold reduction in the infection rate of cells expressing full-length IncA-His compared to non-transfected cells from the same well. This result is in agreement with our previous observation, based on microscopy data, that ectopic IncA expression [21], but not that of the deletion mutant [15], ultimately results in cell death of infected cells. This was not a non-specific consequence of transfection since cells expressing Δ75IncA-His were actually slightly better infected than the non-transfected cells, a trend that we repeatedly observed with transfection of other negative controls (data not shown).

### Quantification of EBs production

**Figure 5:**
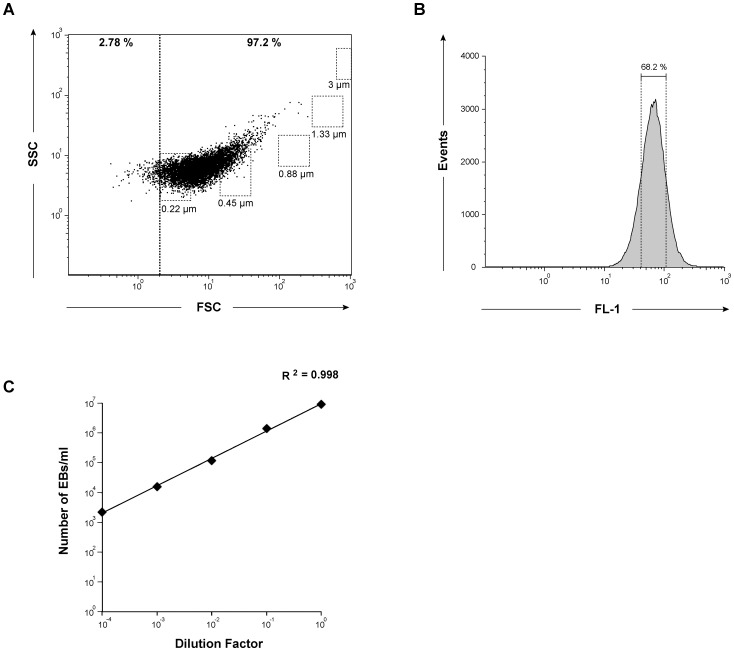
Quantification of EBs by flow cytometry. (A) Dot-plot of the size and granulometry of fluorescent particles in an EB preparation. The threshold of background fluorescence in the green channel was determined using non-infected cells broken with glass beads and fixed in 2% (w/v) PFA. A purified EB preparation fixed in 2% PFA was then acquired, and parameters in the FSC and SSC channels of events above the background green fluorescence threshold are shown. The squares delimit the perimeter of detection of calibration beads of the indicated size. The size of the fluorescent particles detected ranged between 0.22 and 0.45 µm. (B) Fluorescence of the L2^incD^GFP EBs. 68.2 percent (mean±1 standard deviation) of the particles have a fluorescence between 40 and 105, thus reflecting a maximum of 2.5-fold variation in the fluorescence of individual EBs. (C) A fresh EB preparation was serially diluted and fixed in 2% PFA. For each dilution, the concentration of particles in the sample was calculated by flow cytometry based on the fluid speed (ml.min^−1^) and event detection (event.min^−1^).

Having determined that the GFP signal emitted by L2^incD^GFP EBs is detected by microscopy, we asked whether this fluorescence could be used to detect individual EBs using flow cytometry. New generation flow cytometers have lowered the size limit for detection of particles and are suitable for the detection of small particles including bacteria. However, the small size of EBs (around 0.3 µm) makes them particularly challenging to detect by this method. Using uninfected cells to set up the background of fluorescence, we detected fluorescent particles in a sample containing purified L2^incD^GFP EBs that were absent from a preparation of purified L2 EBs and from non-infected cells. When gating on this fluorescent population, and using calibration beads, we observed that the particle size ranged between 0.22 and 0.45 µm, which is the expected size for EBs (Fig. 5A). The distribution of the fluorescence values gives a sharp peak, indicating that the fluorescence of individual EBs was homogenous (Fig. 5B). By calibrating on the fluid speed, we estimated the concentration of serial dilutions of one fresh bacterial preparation with a good approximation over 4 logs, down to 10^3^ EBs/ml (Fig. 5C). The same preparation, determined by flow cytometry to contain 17.7±2×10^6^ EBs/ml, was used to serially infect cells. We found that the sample contained 16.9±4×10^6^ IFU/ml, validating the direct particle enumeration. This result also shows that the particles detected by flow cytometry were infectious.

## Discussion

The L2^incD^GFP strains proved suitable to track the chlamydial developmental cycle by flow cytometry: (i) its growth is identical to that of the parental non-fluorescent strain ([13] and Fig. 1A), (ii) the fluorescence signal can be used as a read-out of bacterial multiplication, (iii) the fluorescent signal was detected in the EBs and allowed for direct quantification of the extracellular and intracellular bacteria.

For the same bacterial load, the fluorescent signal was about three-fold stronger in cells infected with L2^incD^GFP than with L2^nm^GFP at the mid-phase of the cycle (not shown). It likely reflects the fact that the GFP signal expressed by SW2::GFP plasmid is hindered by the fusion of the GFP with chloramphenicol acetyl transferase [11]. It might also reflect a difference in strength of the two promoters or in their timing of activity during the cycle. The *incD* promoter is expressed very early and during the whole developmental cycle [22], which might explain why we could detect bacterial growth even at early time points. Use of other promoters might delay the detection of the fluorescent marker. However, it is not known if the distinction between early and late promoters holds true when the genes are expressed from the plasmid.

Traditionally, effects of antichlamydial compounds on infection are assessed by measuring genome numbers by quantitative PCR or by measuring the resulting IFU through reinfection of fresh cells. These methods are costly, labor intensive, and often only one time point is selected for the analysis. Analysis of infection via flow cytometry is cheaper, faster, more quantitative, especially when analyzing rare events, and gives additional information. Measure of the GFP signal gives immediate access to the percentage of infected cells and to the mean intensity of fluorescence in the infected population. These data are very useful to determine the inhibition properties of antimicrobial compounds. Unlike measures of IFUs or genome numbers, they distinguish between antimicrobial activities that prevent establishment of the infection (fewer infected cells, normal rate of accumulation of GFP in the infected population) from those that prevent bacterial growth (same percentage of infected cells, slower accumulation of GFP). In addition, these parameters can be correlated to a third one such as the expression of a protein in a non-homogenous population of transfected cells, as illustrated with the inhibition of bacterial development in cells expressing full-length IncA. Altogether, these GFP-expressing strains, coupled to high throughput flow cytometry, offer a powerful tool in a wide range of studies, in particular rapid screening of anti-chlamydial compounds. For instance we found that the IC_50_ for tetracycline is 40 ng/ml. This finding is in agreement with a previous report [23]. In addition, we showed that GFP-loaded EBs allow the measurement of bacterial adhesion to cells by flow cytometry, simplifying an alternative procedure using bacteria labeled with a fluorescent dye [4].

Until now the most direct method to measure EB entry was a two-step procedure, in which external and internal bacteria were successively stained before and after permeabilization of the sample [5], [6]. This method, followed with manual counting of internalized bacteria is tedious. Consequently, EB internalization rates are mostly absent from the literature, with, to the best of our knowledge, a single report on the kinetics of *C. pisttaci* entry [24]. In addition, using FITC-coupled bacteria, we had noticed that some of the internalized bacteria were not accessible to antibodies, even after saponin permeabilization, possibly because actin polymerization around nascent inclusions limits antibody access. The green fluorescence of the EBs reduces the time needed to process the sample (one single labeling step), and eliminates underscoring of intracellular bacteria due to limited antibody access. Also, the green fluorescence is strong enough to allow automated detection by the ICY software, which drastically reduces the analysis time. Provided that enough images are collected, the procedure can be applied to very low MOI. It will facilitate future discovery of anti-chlamydial compounds that target the internalization step per se.

Finally, we show here that, in spite of their small size, GFP-expressing EBs can be enumerated by flow cytometers designed for the detection of small particles. This is a huge advantage over current methods of titrating bacterial preparations, which require reinfecting fresh cells to measure IFUs. Using calibrating beads, we determined that the EB diameter was between 0.22 and 0.45 µm, which fits well with observations at the ultrastructural level. We could also observe fluorescent particles between 0.88 and 1.33 µm, the expected size range for RBs (data not shown). However, even when the bacteria were collected at mid-cycle, either in PBS or in the conventional sucrose phosphate glutamate buffer used for chlamydial preparation, we recovered a minority of these larger particles compared to EBs, suggesting that the majority of RBs is lost by lysis during sample preparation.

*C. trachomatis* L2 was the first *Chlamydia* strain engineered to express GFP. Recent work has shown that the same plasmid can be stably inserted in other *C. trachomatis* strains, as well as in *C. pneumoniae* [25], [26]. It is very likely that the quantitative methods we developed here are also applicable to other chlamydial species. The use of GFP-expressing *Chlamydia* will greatly improve the quantitative assessment of the progression of these different chlamydial species throughout their developmental cycles and will aid in the identification of compounds that affect specific steps of the cycle.

## Supporting Information

Figure S1 **Analysis of the early times of** ***C. trachomatis*** **L2 development using flow cytometry.** Cells were infected at a MOI  =  0.3 and fixed at the indicated times. Samples were analyzed by flow cytometry as described in the Methods section, and histograms of fluorescence in the green channel (FL-1) are shown. For each time point, 10,000 cells were analyzed. The horizontal bar delimits the fluorescence above background level. The percentage of cells included in this gate, and their mean fluorescence, are indicated. NI  =  non-infected. For each time point, one coverslip infected in the same conditions was fixed and permeabilized to stain the DNA with Hoechst 33342. DNA appears in blue and GFP-expressing bacteria (arrowheads) in green, bar = 10 µm.
(TIF)
