# Supplementary figures and images for: Quantitative Monitoring of the Chlamydia trachomatis Developmental Cycle Using GFP-Expressing Bacteria, Microscopy and Flow Cytometry
Source: PLoS One. 2014 Jun 9;9(6):e99197. doi: 10.1371/journal.pone.0099197 (PMC4049595; doi:10.1371/journal.pone.0099197)

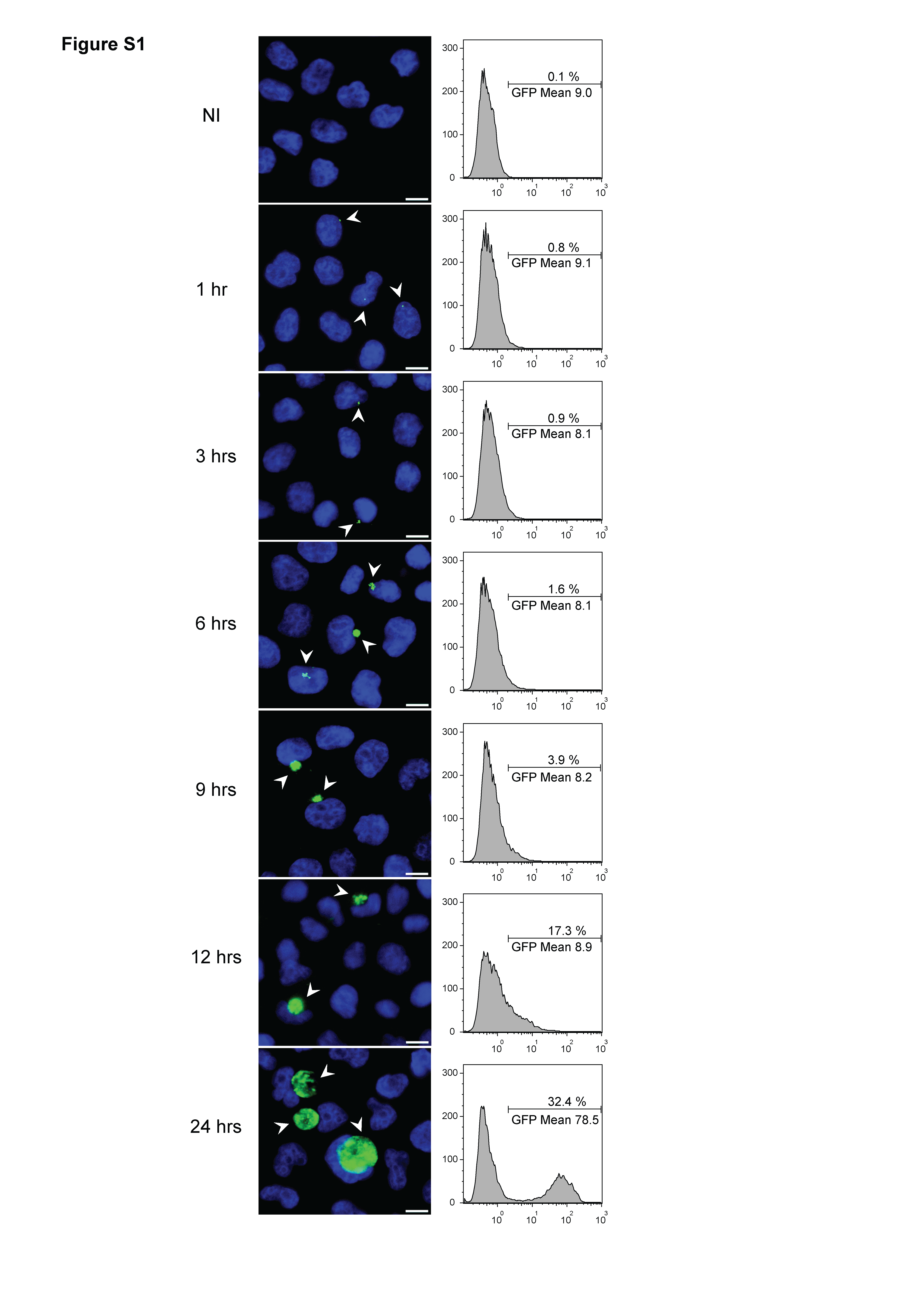

Supplement: Figure S1 — Analysis of the early times of C. trachomatis L2 development using flow cytometry. Cells were infected at a MOI = 0.3 and fixed at the indicated times. Samples were analyzed by flow cytometry as described in the Methods section, and histograms of fluorescence in the green channel (FL-1) are shown. For each time point, 10,000 cells were analyzed. The horizontal bar delimits the fluorescence above background level. The percentage of cells included in this gate, and their mean fluorescence, are indicated. NI = non-infected. For each time point, one coverslip infected in the same conditions was fixed and permeabilized to stain the DNA with Hoechst 33342. DNA appears in blue and GFP-expressing bacteria (arrowheads) in green, bar = 10 µm. (TIF) [file pone.0099197.s001.tif]
